# Meteorological Phenomenon

**Published:** 1853-03

**Authors:** 


### METEOROLOGICAL PHENOMENON.

During a storm of wind and rain, on the night of the 25th of March, a yellow, impalpable powder fell in this city and neighborhood, in such quantities as in some places to cover the ground. Being light, it floated upon the water, and formed a thick film along the sides of the gutters. The phenomenon gains interest from the circumstance that a similar precipitate occurred in Tennessee ten years ago (March, 1843), after a cold, backward spring, much like the present. Then, as recently, it was accompanied by a rain which succeeded a day of high southerly winds. The powder had very much the appearance of *flowers of sulphur*. It was combustible, and burnt with the odor of vegetable matter. The explanation generally given of it is, that it is the pollen of flowers wafted into our region by the winds from the South.

